# Discovering biomarkers from gene expression data for predicting cancer subgroups using neural networks and relational fuzzy clustering

**DOI:** 10.1186/1471-2105-8-5

**Published:** 2007-01-06

**Authors:** Nikhil R Pal, Kripamoy Aguan, Animesh Sharma, Shun-ichi Amari

**Affiliations:** 1 Electronics and Communication Sciences Unit, Indian Statistical Institute, 203, B. T. Road, Calcutta – 700108, India; 2 Neurogenetics Laboratory, RIKEN Brain Science Institute, Saitama, Japan; 3 Department of Computer Science and Electrical Engineering, University of Missouri, Kansas city, USA; 4 Lab for Mathematical Neuroscience, RIKEN Brain Science Institute, Saitama, Japan

## Abstract

**Background:**

The four heterogeneous childhood cancers, neuroblastoma, non-Hodgkin lymphoma, rhabdomyosarcoma, and Ewing sarcoma present a similar histology of small round blue cell tumor (SRBCT) and thus often leads to misdiagnosis. Identification of biomarkers for distinguishing these cancers is a well studied problem. Existing methods typically evaluate each gene separately and do not take into account the nonlinear interaction between genes and the tools that are used to design the diagnostic prediction system. Consequently, more genes are usually identified as necessary for prediction. We propose a general scheme for finding a small set of biomarkers to design a diagnostic system for accurate classification of the cancer subgroups. We use multilayer networks with online gene selection ability and relational fuzzy clustering to identify a small set of biomarkers for accurate classification of the training and blind test cases of a well studied data set.

**Results:**

Our method discerned just seven biomarkers that precisely categorized the four subgroups of cancer both in training and blind samples. For the same problem, others suggested 19–94 genes. These seven biomarkers include three novel genes (NAB2, LSP1 and EHD1 – not identified by others) with distinct class-specific signatures and important role in cancer biology, including cellular proliferation, transendothelial migration and trafficking of MHC class antigens. Interestingly, NAB2 is downregulated in other tumors including Non-Hodgkin lymphoma and Neuroblastoma but we observed moderate to high upregulation in a few cases of Ewing sarcoma and Rabhdomyosarcoma, suggesting that NAB2 might be mutated in these tumors. These genes can discover the subgroups correctly with unsupervised learning, can differentiate non-SRBCT samples and they perform equally well with other machine learning tools including support vector machines. These biomarkers lead to four simple human interpretable rules for the diagnostic task.

**Conclusion:**

Although the proposed method is tested on a SRBCT data set, it is quite general and can be applied to other cancer data sets. Our scheme takes into account the interaction between genes as well as that between genes and the tool and thus is able find a very small set and can discover novel genes. Our findings suggest the possibility of developing specialized microarray chips or use of real-time qPCR assays or antibody based methods such as ELISA and western blot analysis for an easy and low cost diagnosis of the subgroups.

## Background

Gene expression data are ever increasingly being used in the fields of medicine including categorization of cancers into different diagnostic subgroups, which often appear similar in routine histology [1-3]. We focus our attention on one such important problem, proper classification of various groups of childhood cancers, collectively known as *small round blue cell tumors *(SRBCTs). The worldwide incidence rates of childhood cancers vary widely from as high as 155 per million persons in Nigeria to 40 per million persons in the Indian population of Fiji. The incidence rate of childhood cancer in the USA is approximately 125 per million persons [4,5]. Amongst the various childhood cancers, SRBCT is the third most frequently occurring type (18%) and consists of neuroblastoma (NB, 7%), non-Hodgkin lymphoma (NHL, 6%), rhabdomyosarcoma (RMS, 3%), and Ewing sarcoma (EWS, 2%) [4,5]. These heterogeneous types of cancer present a similar histology of small blue tumor cell and thus often leads to misdiagnosis. Accurate diagnosis of these subgroups is important as the treatment options, monitoring the response and prognosis may vary widely between these subgroups. Development of cancer is a complex reorganization and remodeling of multiple cellular pathways affecting thousands of genes. Thus the identification of global gene expression signatures rather than depending on a particular gene marker for a specific type of cancer may be ideally suited in categorizing various subgroups of SRBCTs. Our objective here is to identify a small set of biomarkers to design useful diagnostic prediction systems for accurate classification of the four categories of SRBCTs.

Typically gene expression data suffer from three problems : (i) limited number of available examples, (ii) very high dimensional nature of the data, and (iii) noisy characteristics of the data. Moreover, for a given problem, usually a few genes are required. So we face two challenging tasks: finding a minimal set of genes that has an adequate discriminating power to categorize the subgroups and designing of a prediction system using the selected genes to accurately classify unseen examples. Use of a minimal number of genes is consistent with the principal of *minimum description length *(MDL). Systems designed keeping in mind MDL are likely to yield better generalization (less number of free variables, and hence less likely to result in poor generalization). Moreover, with a small set of genes, it is easy to optimize the diagnostic system better. Model selection can also be done using Akaike Information Criterion (AIC). Several attempts have been made to solve these problems. The different machine learning tools used by researchers include multilayer perceptron (MLP) networks [3], Self-organizing maps [1], nearest centroid classifiers [2], support vector machines (SVMs) [6,7]. Similarly, many gene selection methods have also been used. The gene selection methods often ignore the learning machine used to design the prediction system. Some methods, although, take into account the learning machines, they remove one (or a set of features) at a time in a stepwise manner. Such a method cannot capture the subtle nonlinear interactions between different genes and consequently, one ends up with more genes than what is needed to solve the problem.

To overcome this problem, we use a neural network, which can pick up what is needed (select the required genes) when it learns the diagnostic classification task. This helps the network to honor possible nonlinear interactions between genes. The set of selected genes is further reduced with clustering of correlated genes based on fuzzy sets theory. We apply our method on the same SRBCT data set used by Khan et al. [3] and other researchers [2,6,7]. This data set consists of expression levels of 2,308 genes, which were obtained from glass-slide cDNA microarrays, prepared in accordance with the standard protocol of the National Human Genome Research Institute. We have identified only seven (7) genes that can do the diagnostic classification task with 100% accuracy both on the training data and the blind test data. This set of genes includes three novel genes, NAB2, EHD1, and LSP1, that are not identified by others as important. Our method clearly outperforms other results because for the same data set, the number of marker genes reported by other researchers vary between 19 and 96. Moreover, we have demonstrated that these seven genes perform equally well with other machine learning tools like radial basis function network and support vector machines.

## Results

### Data Set

Khan et al. [3] considered the 2308 genes that passed a filter requiring a minimum expression level and all other processing was done on this set of 2308 genes. We also use the same set of 2308 genes. There are 88 samples of which 63 (EWS:23, NHL:8, NB:12, and RMS:20) samples are used for training. The remaining 25 includes five samples which are later detected to be of non-SRBCT types. Hence for blind testing of the system we use 20 (EWS:6, NHL:3, NB:6, and RMS:5) samples. For this data set, other researchers also used this training-test partition. The five non-SRBCT samples include 2 normal muscle tissues (Sample Nos. 1 and 6, Table 1) and 3 cell lines including an undifferentiated sarcoma (Sample No. 3, Table 1), osteosarcoma (Sample No. 7, Table 1) and a prostate carcinoma (Sample No. 2, Table 1). The data set is available at [8].

**Table 1:** Network outputs for 25 blind test samples

**Sample #**	**EWS**	**RMS**	**BL**	**NB**	**Actual**
1	0.999	0.001	0.000	0.000	Sk. Muscle
2	0.000	0.224	0.000	0.004	Prostate Cancer
3	1.000	0.204	0.000	0.000	Sarcoma
4	0.000	0.000	0.000	1.000	NB
5	0.151	0.157	0.000	0.000	RMS
6	0.039	0.000	0.992	0.000	Sk. Muscle
7	0.016	0.000	0.999	0.000	Osteosarcoma
8	0.000	0.000	0.000	1.000	NB
9	1.000	0.001	0.000	0.008	EWS
10	0.000	1.000	0.000	0.000	RMS
11	0.003	0.000	1.000	0.000	BL
12	1.000	0.000	0.000	0.000	EWS
13	0.000	0.997	0.000	0.000	RMS
14	1.000	0.000	0.000	0.000	EWS
15	1.000	0.001	0.000	0.000	EWS
16	0.309	0.028	0.001	0.000	EWS
17	0.000	1.000	0.000	0.000	RMS
18	0.001	0.000	1.000	0.000	BL
19	0.000	1.000	0.000	0.000	RMS
20	0.000	0.000	0.000	1.000	NB
21	0.000	0.000	0.000	1.000	NB
22	0.000	0.000	0.000	1.000	NB
23	0.000	0.000	0.000	1.000	NB
24	0.000	0.000	1.000	0.000	BL
25	1.000	0.000	0.000	0.000	EWS

The predicted and the actual outputs of a typical run for the 25 blind test samples. These 25 also includes the 5 nonSRBCT cases as marked in column 6 (sample numbers : 1,2,3,6, and 7). All but sample No. 16 of the 25 SRBCT test samples, the support provided by the network for the correct class is almost 1 and for the other classes it is practically 0. For sample No. 16, the support for the correct class is 0.309 but it is about 12 times stronger than the next high output value. Hence, this is also a decision with high confidence. For sample No. 5, although the network classified it correctly, the support for RMS and EWS are 0.157 and 0.151 suggesting further investigation (weak support for the RMS and the next higher support is for EWS, but that is very close to that of RMS.). Due to nonavailability of the patient's identity, this investigation could not be pursued further.

### Neural Networks with online Gene Selection Ability Select Good Biomarkers

We have used a modified multilayered perceptron (MLP) network [9] with online feature selection capability for identification of biomarkers. We call it Feature Selection MLP (FSMLP). Conceptually, each input node (hence each gene) of the FSMLP has a gate associated with it. At the beginning of training, these gates are kept almost closed, and the learning algorithm opens the required gates (allows genes to enter the network) depending on the ability of genes to reduce the training error. This is our first stage. In this stage, we have selected only twenty genes based on the importance of genes as defined by the *gate opening values *(see Materials and Methods). For the FSMLP network to reduce the chances of bad generalization, we have used just one hidden layer with 150 nodes. The set of selected twenty genes are listed in Table 2. These twenty genes have enough characteristic signatures to discriminate the four categories of tumors with 100% accuracy both in the training and test data sets. With these twenty genes, we have trained neural networks 20 times with different initializations, and in each case the system is found to achieve 100% accuracy on the training data as well as on the twenty blind test data.

**Table 2:** The list of twenty best genes selected by FSMLP in the first stage

Gene ID	Image ID	Name
FGFR4	(*)784224	fibroblast growth factor receptor 4
EST	208699	EST
FCGRT	(*)770394	Fc fragment of IgG, receptor, transporter, alpha
AF1Q	(*)812105	transmembrane protein
HCLS1	(*)767183	hematopoietic cell-specific Lyn substrate 1
NAB2	(*)770868	NGFI-A binding protein 2 (ERG1 binding protein 2)
CDH2	(*)325182	cadherin 2, N-cadherin (neuronal)
EHD1	(*)745019	EH domain containing 1
HLA-DQA1	80109	major histocompatibility complex, class II, DQ alpha 1
LGALS3BP	811000	lectin, galactoside-binding, soluble, 3 binding protein (galectin 6 binding protein)
BAT3	898237	HLA-B associated transcript-3
SGCA	796258	sarcoglycan, alpha (50 kD dystrophin-associated glycoprotein)
ESTs	244618	ESTs
NOE1	52076	olfactomedinrelated ER localized protein
LSP1	(*)143306	lymphocyte-specific protein 1
IFG2	296448	insulin-like growth factor 2 (somatomedin A)
PMS2L12	(*)878652	postmeiotic segregation increased 2-like 12
NA	450152	NA
FVT1	(*)814260	follicular lymphoma variant translocation 1
CCNE1	68950	cyclin E1

We used a network with 2308 input nodes, 150 hidden nodes and 4 output nodes. These twenty genes are selected based on the gate opening values. We made several runs of ordinary MLP using these twenty genes and in each case the network was able to correctly classify all training and blind test examples. In the second stage, the FSMLP network is used to select ten best genes from amongst the twenty genes selected in the first stage. These ten genes are marked by asterisks. This set of ten genes has adequate cancer specific signatures to categorize the four types of SRBCTs.

### Relational Fuzzy Clustering and Neural Networks Together Select Only Seven Biomarkers

In order to further reduce the number of biomarkers, we have proceeded as follows: Again we have used the FSMLP to select the best ten marker genes from among the selected twenty genes. We have ensured with repeated trials that these ten biomarkers {CDH2, FGFR4, EHD1, LSP1, FVT1, FCGRT, NAB2, AF1Q, PMS2L12, HCLS1} can do the intended job of classifying both training and blind test data with 100% accuracy. These ten genes are marked with asterisk in Table 2. We have then used the non-Euclidean relational fuzzy c-means (NERFCM) clustering algorithm [10] to cluster the twenty selected genes (not the samples). We have not used Euclidean *distance *to generate the dissimilarity relation for NERFCM because our objective is to eliminate positively correlated genes, if any, in the selected genes. Note that, two highly correlated genes may have a higher distance than two uncorrelated genes. The dissimilarity relation, R, to be used for clustering is computed as a scaled version of Pearson correlation coefficient matrix. The NERFCM algorithm is used to cluster this R into 6 clusters. The algorithm was run several times with different initializations. Six subsets of genes are found to form consistently strong (evaluated in terms of membership value (see Materials and Methods) of belonging to a cluster) clusters: {CDH2, AF1Q, PMS2L12, the gene with Image ID 450152}, {EST with Image ID 208699, HCLS1, EHD1, HLA-DQA1}, {LSP1, IGF2, CCNE1}, {NAB2, LGALS3BP, BAT3, NOE1}, {FGFR4, SGCA, EST with Image ID 244618} and {FCGRT, FVT1}. This partition is found to be very consistent between different runs of the algorithm. We have also experimented with five clusters. Typically, when NERFCM is used to find 5 clusters, the fourth and sixth clusters listed above are merged together. The following analysis reveals that with 6 clusters, the selection of genes becomes easy. For the first cluster the gene with Image ID 450152 is not in the list of selected ten and the gene PMS2L12 has the least gate opening in the list of ten genes, so we have removed both from the list. We have retained both CDH2 and AF1Q because their associated gates were significantly opened and the gate opening values were quite close. For the second cluster, the gene EST with Image ID 208699 and gene HLA-DQA1 are not in the top ten and between HCLS1 and EHD1, gene HCLS1 has a very low importance in terms of gate opening. So we have retained only EHD1. From the third cluster we have retained only LSP1 because the other two genes, IGF2, CCNE1, are not in the list of top 10. Similarly, from the fourth cluster we have dropped three genes which are not in the list of top ten and we are left with only NAB2. From cluster five we have selected only gene FGFR4 as the other two are not in the list of top ten. The last cluster has two genes FCGRT, FVT1 both of which have made their positions in the top ten. Although the gate opening values for both of them are high, their difference is also reasonably high. So, we have retained only gene FVT1 having the higher gate opening value. This brings the list of biomarkers to only 7 (Table 3). These seven biomarkers can discriminate the four groups of tumors and we can design a neural network, which can categorize the training data as well as all blind test samples with 100% accuracy. The consistency of the selected genes has been further established through extensive experiments (see Discussion). Through in-silico experiments, we have demonstrated that these seven genes form a necessary and sufficient set for accurate categorization. Note that, because of the existence of correlated genes, this may not necessarily be the only possible such set. It is worth noting here that for the same data set, Khan et al. [3] reported 96 genes (Table 4), while Tibshirani et al. [2] came up with 43 genes that are required for accurate categorization of the SRBCT groups. Best result found in the literature suggests the need for at least 19 biomarkers [7].

**Table 3:** The list of seven biomarkers selected using the relational fuzzy clustering

Gene ID	Image ID	Name	Rank
FGFR4	784224	fibroblast growth factor receptor 4	2
AF1Q	812105	transmembrane protein	6
NAB2	770868	NGFI-A binding protein 2 (ERG1 binding protein 2)	7
CDH2	325182	cadherin 2, N-cadherin (neuronal)	1
EHD1	745019	EH domain containing 1	3
LSP1	143306	lymphocyte-specific protein 1	4
FVT1	814260	follicular lymphoma variant translocation 1	5

The NERFCM algorithm is used to find 6 clusters in the 20 genes selected in the first stage of the scheme. The partition obtained by the relational clustering and the results of stage two are combined to select just 7 marker Genes.

**Table 4:** Top 96 Genes reported in Khan et al. (2001)

Rank	Image ID	Gene ID	Rank	Image ID	Gene ID
1	296448	IGF2	49	788107	AMPHL
2	207274	IGF2	50	784593	EST
3	841641	CCND1	51	756556	C1NH
4	365826	GAS1	52	208718	ANXA1
5	486787	CNN3	53	308231	EST
6	770394	FCGRT	54	486110	PFN2
7	244618	EST	55	21652	CTNNA1
8	233721	IGFBP2	56	377671	ITGA7
9	43733	GYG2	57	745343	REG1A
10	295985	EST	58	241412	ELF1
11	629896	MAP1B	59	504791	GSTA4
12	840942	HLA-DPB1	60	841620	DPYSL2
13	80109	HLA-DQA1	61	859359	PIG3
14	41591	MN1	62	45542	IGFBP5
15	866702	PTPN13	63	80338	SELENBP1
16	357031	TNFAIP6	64	45291	DRPLA
17	782503	EST	65	323371	APP
18	377461	CAV1	66	897788	PTPRF
19	52076	NOE1	67	377731	GSTM5
20	811000	LGALS3BP	68	784224	FGFR4
21	308163	EST	69	293500	EST
22	812105	AF1Q	70	767183	HCLS1
23	183337	HLA-DMA	71	297392	MT1L
24	714453	IL4R	72	325182	CDH2
25	298062	TNNT2	73	1435862	MIC2
26	39093	MNPEP	74	377048	EST
27	212542	EST	75	814260	FVT1
28	204545	EST	76	784257	KIF3C
29	383188	RCV1	77	42558	GATM
30	82225	SFRP1	78	814526	HSRNASEB
31	44563	GAP43	79	839736	CRYAB
32	289645	APLP1	80	395708	DPYSL4
33	324494	HSPB2	81	416959	NFIB
34	563673	ATQ1	82	364934	DAPK1
35	1473131	TLE2	83	868304	ACTA2
36	1416782	CKB	84	755599	IFI17
37	417226	MYC	85	246377	EST
38	878280	CRMP1	86	291756	TUBB5
39	812965	MYC	87	809901	COL15A1
40	122159	COL3A1	88	769959	COL4A2
41	609663	PRKAR2B	89	796258	SGCA
42	461425	MYL4	90	854899	DUSP6
43	1469292	PIM2	91	755750	NME2
44	809910	1-8U	92	292522	EST
45	824602	IFI16	93	308497	EST
46	245330	IGF2	94	813266	FHL1
47	135688	GATA2	95	200814	MME
48	1409509	TNNT1	96	768370	TIMP3

List of 96 genes selected by Khan et al. using a neural network based method.

### The identified Biomarkers are Important in Cancer Biology

**Figure 1:**
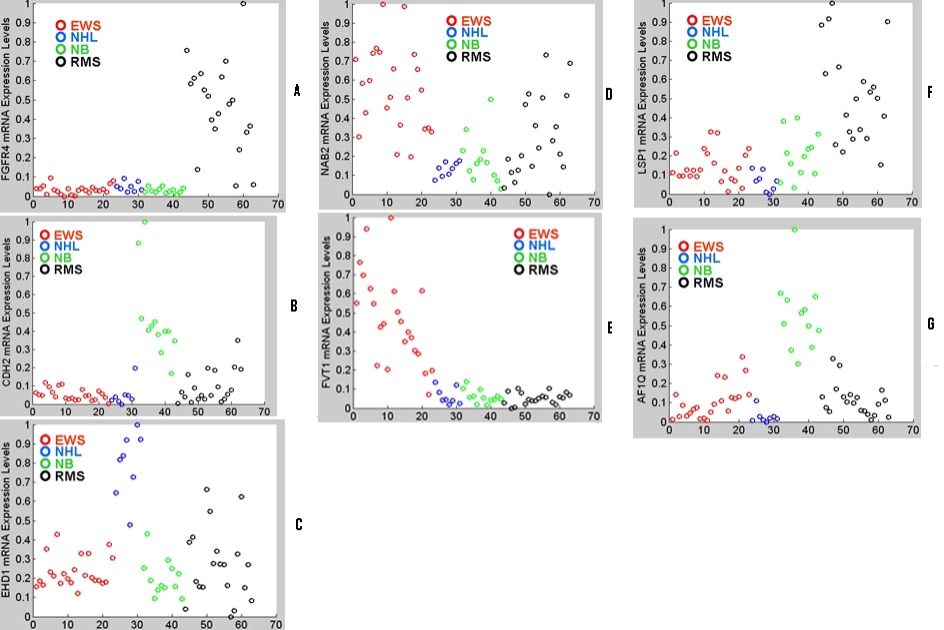
**Scatterplot of expression values of the 7 genes in the training set**. Each panel corresponds to one gene. The red, blue, green and black colors respectively correspond to EWS, NHL, NB and the RMS type of SRBCTs. (A) FGFR4 is upregulated only for RMS, (B) CDH2 is upregulated only for NB (C)EHD1 is moderate to highly expressed for NHL and RMS (D) NAB2 is moderate to highly expressed for EWS and RMS (E) FVT1 is upregulated only for EWS (F) LSP1 is moderate to highly expressed only for RMS (G) AF1Q is moderate to highly expressed for NB.

The set of seven genes that our system identified is involved in the biological process of cancer. For example, this set of seven genes includes an interesting gene NAB2 (EGR1 binding protein 2) which neither Khan et al. [3] and nor Fu & Fu-Liu [7] had found important. Typically, this gene is downregulated in various tumors. NAB2 is a corepressor of EGR (early growth response gene) and its expression depends on tumor types and stages. For example, NAB2 is often downregulated in prostate cancer but upregulated in malignant melanoma [11,12]. In our analysis we observed that NAB2 is moderate to highly upregulated in EWS and in a few cases of RMS; while for the NHL and NB cases it is practically absent (see Figure 1D). Interestingly, a search in GEO profiles (Gene Expression Omnibus, NCBI) also showed a moderate expression of NAB2 in few cases of Ewing sarcoma (GPL1977 1934). Thus, not only the involvement of NAB2 in tumorigenesis but also its distinct signature in various types of tumors are singled out by our analysis.

EH domain containing 1 (EHD1) is another novel gene in our list of biomarkers which others could not find important [3,7]. EHD1 protein is involved in endocytosis and trafficking of various membrane protein including MHC class proteins, insulin-growth factors and secretion of glucose transporter 4 (GLTU-4) [13]. Although EHD1 has not been studied in the context of cancer biology, it seems to be highly expressed in metastatic colon cancer than in tumor of the colon as per the GEO profiles (GEO: GPL96 208112). However, in EWS (GEO: GPL1977 1465), breast tumor (GEO: GPL180 3948) and in B-cell lymphoma (GEO: GPL176 5453) the gene expression is downregulated but slightly upregulated in RMS (GEO: GPL1977 1465). We observed that EHD1 upregulated in Non-Hodgkin Lymphoma (NHL) and in a few cases of RMS; while in a majority of RMS and EWS cases it is moderately expressed.

CDH2 belongs to the family of cell-cell adhesion molecules and mostly their reduced expression leads to tumor invasiveness [14]. Loss of or impaired cell adhesion are important determinants in epithelial neoplasia [15]. In pancreatic cancer CDH2 expression is silenced [16]. We observed that CDH2 expression is practically absent in the EWS and NHL groups of tumors, while for the NB class its expression varied from moderate to very high levels. For a few RMS cases also it is found to be moderately expressed. This might be an indicator that plausibly CDH2 had either acquired mutation or protein truncation in RMS. Also a search in the GEO profiles showed that CDH2 is largely downregulated in EWS (GPL1977 7918). Therefore, it seems that CDH2 expression is tumor specific. And in case of SRBCT family of tumors CDH2 provides us a distinctive signature for categorizing the various tumor classes.

A fourth relevant gene inferred by our system to have class-specific signature is fibroblast growth factor receptor 4 (FGFR4). This tyrosine kinase receptor binds to fibroblast growth factor, a mitogenic ligand, and carry out the signal transduction to the intracellular environment in cellular proliferation, differentiation and migration [17]. In normal tissues, FGFR4 expression is hardly detectable. However, overexpression of FGFR4 has been shown in various cancers, including pituitary, prostate, thyroid [18-20]. In these cases either mutation in FGFR4 (Gly338Arg) or truncation in its protein was involved resulting in deregulated FGFR4 mediated signaling. However, in lung adenoarcinoma, FGFR4 is downregulated [21]. Curation from GEO profiles of NCBI also supports our observation that shows downregulation of FGFR4 in EWS but moderate expression in RMS (GPL1977 11439). We observed that for the RMS group, it is significantly upregulated but for NB, EWS and NHL groups the FGFR4 expression is practically absent revealing a remarkable RMS-specific signature.

The gene LSP1 is involved in transendothelial migration of neutrophil and actin cytoskeleton organization through MEK1 and ERK2 pathways [22,23]. We observed downregulation of LSP1 in EWS, NHL and NB but reasonably higher expression in the RMS group of tumors. Interestingly, in diffuse large B-cell lymphoma (DLBL) LSP1 is also very much downregulated (GEO: GPL176 13667). However, LSP1 expression has been found to increase in NHL class of B-cell lymphoma [24]. Thus, LSP1 regulation is tumor class specific and useful for subcategorization of tumors.

The ALL1-fused gene from chromosome 1q (AF1Q) is known to play important roles in leukemia. In particular, it was detected as a mixed-lineage leukemia (MLL) fusion partner from infant acute myelomonocytic leukemia carrying the t(1;11)(q21;q23) translocation [25]. This MLL fusion partner also plays an important role in acute myeloid leukemia (AML). The expression level of AF1Q is shown to be correlated with the clinical outcome in pediatric patients with AML. The elevated expression of AF1Q is found to be an independent adverse prognostic factor in pediatric AML [26]. Further AF1Q is found to be expressed in high metastatic potential breast cancer cells rather than low metastatic potential breast cancer cells and overexpression of AF1Q in the later cell renders it highly metastatic [27]. Thus AF1Q plays important roles in different types of cancer. We found that AF1Q is very much downregulated for the NHL, EWS and RMS groups of tumors and is moderate to highly upregulated for the neuroblastoma group.

The gene FVT1 also an important one with cancer specific characteristic. Its expression is practically absent for RMS, NB and NHL groups of tumors, while for the EWS group it is highly expressed signifying a very distinct tumor-specific signature.

### The Seven Biomarkers Can Detect Non-SRBCT samples

**Figure 2:**
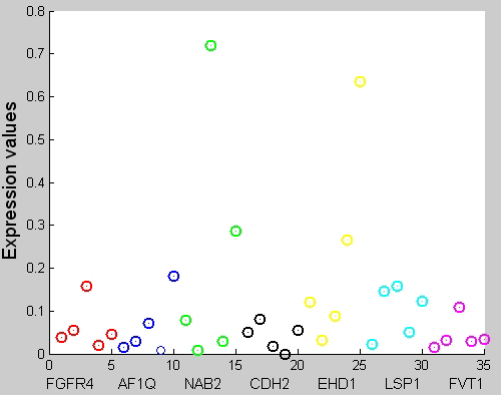
**Scatterplot of the 7 genes for the 5 nonSRBCT samples**. The 7 groups (each plotted with a different color) correspond to seven selected genes { FGFR4, AF1Q, NAB2, CDH2, EHD1, LSP1, FVT1} in order. Each group has five components one corresponding to each of the 5 non-SRBCT test cases. The test cases appear in this order : Skeletal Muscle tissue (test case 9, normal), Prostate carcinoma (test case 11, cancer), undifferentiated sarcoma (test case 5, cancer), Skeletal Muscle tissue (test case 13, normal) and osteosarcoma (test case 3, cancer). The Scatterplot shows that for the test case 5 (sarcoma)only NAB2 is highly expressed. For the test case 3 (osteosarcoma), EHD1 is upregulated along with moderately expressed NAB2. This is not typical of the SRBCT cases.

**Figure 3:**
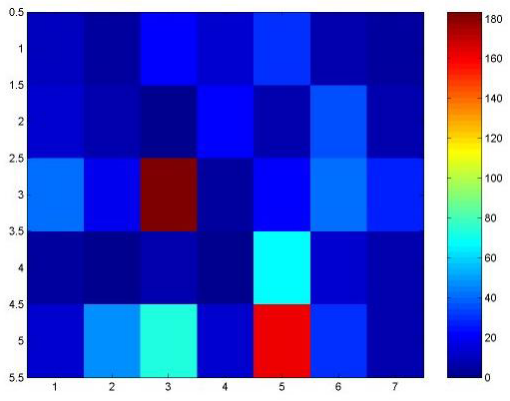
**Pseudo color image of the 7 genes for the 5 nonSRBCT samples**. The dark blue to dark red colors correspond to low to high expression values. For each of test cases 5 and 3 the expression level of only one gene is high, for all other genes and cases the expression levels are quite low.

In the original SRBCT data set [3] there were five non-SRBCT samples : two normal muscle tissues, three cell lines consisting of an undifferentiated sarcoma, an osteosarcoma, and a prostate carcinoma. It is surprising to note that for these five non-SRBCT examples almost all of the seven genes are downregulated (see Figure 2). Although, we did not use any non-SRBCT examples to decide on the biomarkers, our genes can detect non-SRBCT examples, at least for four samples in Figure 2. Figure 2 has seven groups, each plotted using a different color. The seven groups correspond to seven selected genes { FGFR4, AF1Q, NAB2, CDH2, EHD1, LSP1, FVT1} in order. Each group has five components one corresponding to each of the 5 non-SRBCT test cases. The test cases appear in this order : Skeletal Muscle tissue (test case 9, normal), Prostate carcinoma (test case 11, cancer), undifferentiated sarcoma (test case 5, cancer), Skeletal Muscle tissue (test case 13, normal) and osteosarcoma (test case 3, cancer). Figure 2 shows that for the test case 5 (sarcoma) only NAB2 is highly expressed. This example may be confused as Ewing Sarcoma. For the test case 3 (osteosarcoma), EHD1 is upregulated along with moderately expressed NAB2. This is not typical of the SRBCT classes. The upregulation of EHD1 and NAB2 clearly reveals the cancer-specific characteristic signatures of the identified genes. This is also revealed by Figure 3, which displays the expression levels of different genes as an image for the five outliers.

### Simpler techniques may be used for easy diagnosis of SRBCTs with visual inspection

**Figure 4:**
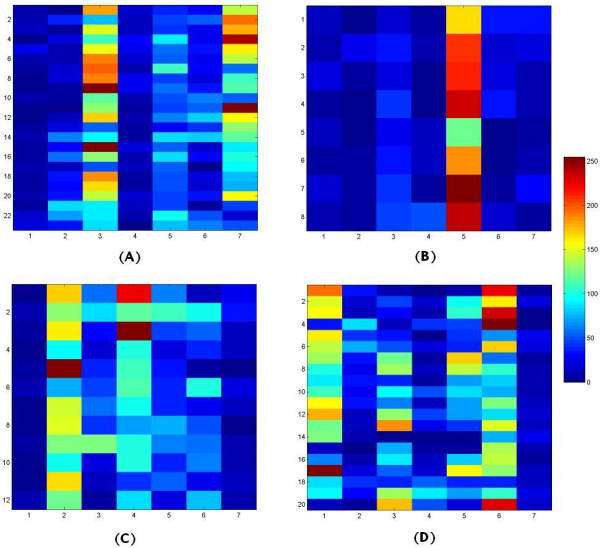
**Pseudo color image of the training data for the 4 SRBCT classes**. Each of the 4 panels represents one class. The dark blue to dark red colors correspond to low to high expression values. (A) EWS : The image reveals that moderate to high upregulation of NAB2 or FVT1 and downregulation of other five genes can signal EWS. (B)NHL : It suggests that a very high upregulation of EHD1 and downregulation of other six genes are signatures of NHL. (C) NB: Moderate to high upregulation of AF1Q and CDH2 and downregulation of FGFR4 and FVT1 indicate the presence of NB. (D)RMS: Upregulation of FGFR 4 or LSP1 and downregulation of the FVT1 are indicator of RMS

Figure 4 has four panels, each displaying the expression values of the training samples of a particular class. Comparison of these four panels reveals four simple approximate diagnostic rules, one for each of the four SRBCT groups:

EWS : Moderate to high upregulation of NAB2 or FVT1 and downregulation of other five genes.

NHL : Very high upregulation of EHD1 and downregulation of other six genes.

NB : Moderate to high upregulation of AF1Q and CDH2 and downregulation of FGFR4 and FVT1.

RMS : Upregulation of FGFR 4 or LSP1 and downregulation of the FVT1.

Note that, in these (approximate) rules, the upregulation of a particular gene is associated with only one rule or group. This mutual exclusion further suggests that the set of identified biomarkers are essential.

Figure 4 and the above rules bespeak the possibility of developing simpler and low cost methods for an easy diagnosis of the subgroups. For example, based on antibodies of the respective protein products of the genes we can use western blot analysis or Enzyme-Linked ImmunoadSorbent Assay (ELISA) to classify the SRBCT samples. One can also use real-time qPCR assays.

Another possibility would be to design specialized microarray chips. For example, one may design microarray chips only for these seven genes with replication. More specifically, chips with 7 × *T *probes, where each row will represent *T *probes for only one of the seven genes. Thus, the expression level of each gene will be replicated *T *times in a row. This replication will make the visual assessment easy and will help to eliminate the effect of noise that is typically encountered in gene expression values. We think that (T =) 6–7 times replication of each probe would be enough for visual inspection because human eyes can easily perceive the contrast between lines with thickness of 6–7 pixels.

### The identified Biomarkers are Universal in Nature (Essential and Sufficient)

We want to emphasize that typically the discriminating ability of a feature or gene should be evaluated keeping in mind the machine learning tool that will be used to design the diagnostic system because the best set of genes for a neural network may not necessarily be the best for a decision tree or for nearest centroids classifiers. On the other hand, a set of good biomarkers should be able to do a good job of prediction using different machine learning tools. Thus, if the selected set is essential and sufficient then it should have "universal" characteristics and hence should be able to do a good job with different tools. To assess this universal character, we evaluated the seven genes with RBF net [28], support vector machines (SVMs) [29], and the *nearest centroid classifier*. The RBF net with 12 Gaussian nodes, each with a very low spread (0.65), can classify all 63 training examples as well as all 20 blind test examples correctly. Note, that we did not optimize the net as each RBF node used the same spread. So, the set has an adequate discriminating power even for RBF network – this is a very desirable feature of good biomarkers. A RBF network even with just *four *nodes, one for each class, can classify all training examples correctly and makes two mistakes on the blind test data; while a RBF network with only five Gaussian nodes, each with a very low spread of 0.5 (i.e., high specificity) can classify all but one test example of the blind test data.

Support vector machines finds a separating hyperplane between two classes either in the original input space or in some high dimensional projected feature space. For this four-class problem, we used one-vs-one (OVA) strategy which trains one classifier (SVM) for each pair of classes. Then voting is used to decide the class label of a data point. Use of Gaussian (RBF) kernels with a very low spread for all SVMs results in zero error both on the training data as well as on the unseen test data. It is worth mentioning here that Fu and Fu-Liu [7] required *nineteen *(19) genes selected using and for SVMs to achieve zero training and test errors. This reconfirms the usefulness and universal characteristics of the identified biomarkers. Note that, we use the word "essential and sufficient" with respect to a set as a whole and hence there could be other such sets. Like any other data driven approach we assume that the training data set is representative of the four categories.

**Figure 5:**
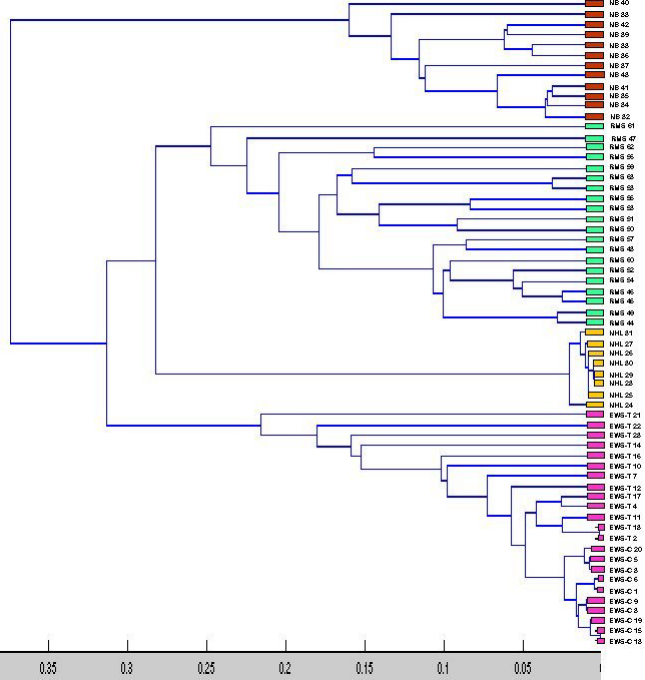
**Dendrogram of the 63 samples in the training data set**. The dendogram is obtained by the single linkage clustering algorithm. We have used the Pearson's correlation matrix computed using only the selected seven genes. If we cut the dendrogram for 4 clusters, training data from different groups are found to form separate clusters as shown by different colors.

We have also analyzed the seven genes using unsupervised learning. In particular, we used the single linkage clustering algorithm to cluster the relation (**1**-R), where R is the Pearson's correlation matrix between the samples, each sample is treated as a sequence and **1 **is a 63 × 63 matrix with each entry equal to unity (1). The four natural *groups *(clusters) found by single linkage algorithm match exactly with the four classes of the SRBCT (see figure 5).

## Discussion

We have identified a set of just seven genes using an online feature selection method based on neural networks and designed a diagnostic system that can classify both the training and unseen test examples with 100% accuracy. To establish that the selected marker genes indeed constitute a minimal set, we made 20 runs of for each possible subset of six genes from the set of selected genes. The networks are trained with the 63 training data points and tested with the same blind set of 20 examples. For each subset of six genes we report the maximum, minimum and the average number of misclassifications on the training as well as on the blind test data in Table 5.

**Table 5:** Training and test error statistics with all possible subsets of six biomarkers

	Training Error in %			Test Error in %		
Gene Removed	Max.	Min.	Average	Max.	Min.	Average

FGFR4	87.30	0	18.18	85.0	15.0	37.0
AF1Q	68.25	0	15.71	80.0	10.0	34.5
NAB2	87.30	0	20.24	85.0	15.0	37.25
CDH2	68.25	0	30.32	75.0	10.0	43.0
EHD1	68.25	0	20.56	75.0	10.0	32.75
LSP1	68.25	0	11.59	70.0	10.0	26.25
FVT1	68.25	0	10.40	75.0	0.0	20.5

The maximum, minimum and the average training and test errors obtained using MLP networks with all possible subsets of size six genes (i.e., after removing one gene at a time from the set of identified marker genes). With each combination of 6 genes, the network was trained 20 times with different initializations and the statistics are computed based on the results of these 20 trials

Of the 140 trials, only in *one *run the error on the blind test data was zero. The average test error is quite high, the minimum value of the average test error rate is more than 20%. Similarly, for the training data also the minimum value of the average training error is more than 10%. This suggests that if we remove any other gene from this set, we shall not be able to distinguish between the four categories of tumors.

To find how confident our network is in making decisions, we analyzed the output of a typical run of network for the blind test samples. For each example, each output node of the network produces a value between 0 and 1. Except for two blind test examples, the network produced almost crisp output (full confidence of 1 only for the correct class and for other classes it is practically zero, see Table 1. For test case 16, although the output was not crisp but the strength for the correct class is about 12 times more than that of its closest competitor, and hence this is a prediction with a high confidence too. The test example 5, which belongs to RMS, although is correctly classified by the MLP, it suggests a good resemblance with the EWS tumor group (The network outputs for the two classes are very close, 0.151 for EWS and 0.157 for RMS). This signals that this case should be looked at more carefully, probably we should look for further information. Due to nonavailability of the patient's identity, we could not make a follow up study on it. This test case is also one of the two cases that are confused by the nearest centroids classifier. A RBF network with just five Gaussian nodes can yield zero training error and can classify correctly all but this example in the blind test set.

In order to make a fair comparison of performance of our method with other methods in the literature, we have used the same training-test partition as used by others and like others achieved 100% training and test accuracies. It may not always be possible to achieve such a high accuracy with classification of other types of cancer or even with new data sets for the same problem. In general, to avoid the dependency of the classifier on the particular training data set used (in other words, to reduce the variance part of the classification error), one should use multiple classifiers and then combine the outputs of the multiple classifiers. Ensemble classification methods such as bagging [30], boosting [31,32] help us to reduce the classification error by reducing the variance.

Khan et al. [3] used PCA and then used a single layer feed-forward network. They analyzed the sensitivity of the network output with respect to changes in the expression level (input) and used this information to rank the genes. They used 96 top ranked genes (see Table 4) because the training error was reduced to zero with 96 genes. We think, one of the reasons their method ended up having so many genes is that it used a single layer network that cannot capture nonlinear boundaries. The other reason may be that their gene selection method looked at one gene at a time and hence could not exploit possible subtle nonlinear interactions between genes. We have demonstrated that the same task can be done using the same tool with just seven marker genes. These seven genes are equally effective with other machine learning tools also. Of these seven genes only four are included in the list identified by Khan et al.

Tibshirani et al. [2] used a nearest centroid method with shrunken centroids. The nearest shrunken centroids method identifies subsets of genes that best characterize each class. This method shrinks the centroid of each class towards the overall centroid using the within-class standard deviation of each gene. A higher importance is given to a gene whose expression is stable within samples from the same class. As a result, many genes are effectively eliminated from the centroids. The shrunken centroid method yielded 43 genes that include only four of the genes identified by us.

Ramaswamy et al. [6] used support vector machine (SVM) for gene selection and multi-class cancer diagnosis. Recently, Fu and Fu-Liu [7] applied the method of Ramaswamy et al. on the SRBCT data set and could find a set of 8 genes that can yield zero training error but 90% accuracy on the blind test data.

Then Fu and Fu-Liu [7] proposed a modified method based on SVM for gene selection and classification. This method is also iterative in nature that finds the least important feature, eliminates it and reevaluates the rest. Using the SRBCT data set, they came up with a set of nineteen (19) genes that can achieve 100% accuracy both on the training data and on the unseen test data samples. This list of 19 genes includes four of the marker genes identified by our method.

Of the seven genes that we found, only four (FVT1, CDH2, FGFR4, AF1Q) are included in the set of genes identified by Kahn et al. [3], Tibshirani et al. [2] and Fu and Fu-Liu [7]. The role of these four genes in cancer has been well demonstrated [3,15,16,18-20,25]. Expressions of the remaining three genes (NAB2, EHD1 and LSP1) are either upregulated or downregulated in the various tumors and also in SRBCTs depending on the tumor subgroups [11,12,24]. It is worthwhile to discuss the role of these three genes in cancer biology. Cancer is a sequential process, involving breaking off cells from the primary tumor sites, migration through bloodstream (transendothelial migration), and setting in new places of the organs. NAB2 is a corepressor of transcription factor family EGR (early growth response gene) and inhibits cellular proliferation and growth. Dysregulation of NAB2 will involve unregulated activity of EGR resulting in tumor growth [11,12]. The primary role of EHD1 is endocytosis and protein trafficking such as MHC class molecules that participate in antigen presentation and destruction of abnormal cells [13]. Therefore, aberrant regulation of EHD1 likely would give rise to tumors. The gene LSP1, on the other hand, plays an important part in transendothelial migration of tumor cells in the bloodstream and thus helps in cancer development [22,23]. It, therefore, appears that these three genes are involved in the tumor and cancer progression pathways. This does not necessarily mean that these genes will have the same discriminating power for all types of cancers. In order to find the best set of discriminating biomarkers for different cancers, one needs to use our scheme to analyze data on those types of cancer.

Recently, Lee et al. [33] made an extensive study to compare three feature selection methods in conjunction with eleven classification schemes. These 11 methods include six classical methods like k-nearest neighbor, Fisher's linear discriminant analysis and five tree methods such as CART, Bagging, Boosting. Lee et al. used 50 top ranked genes to evaluate the performance of the 11 classifiers. The average accuracy on the test examples by the five classical methods varied between 11% to 37% while that for the tree methods varied between 1% to 37%. Considering the performance of classifiers and the fact that authors used 50 genes, it is reasonable to conclude that all selected features were not biomarkers.

## Conclusion

We proposed a computational intelligence based scheme for identification marker genes for distinguishing cancer subgroups and tested it on a well known data set on small round blue cell tumors. All methods that we have discussed identified between 19–96 biomarkers to classify the training and test data with 100% accuracy, while our method found only seven genes to do the same task with neural networks. These seven genes include three novel genes which are not found by other researchers. The main reason for this is that our method (see Methods) looks at all genes together and picks up whatever is needed. The relational fuzzy clustering has helped to reduce the number of correlated genes. The genes identified by us are equally good with other tools like RBF network and SVM, although they were identified keeping in view an MLP. Even unsupervised clustering using these seven genes can discover the actual class structure. These seven genes bear distinct cancer specific attributes and as a group plays important roles in cancer biology. In this investigation although we have analyzed the SRBCTs, the proposed methodology can be used for knowledge discovery related to other diseases

## Methods

### Outline of the Method

**Figure 6:**
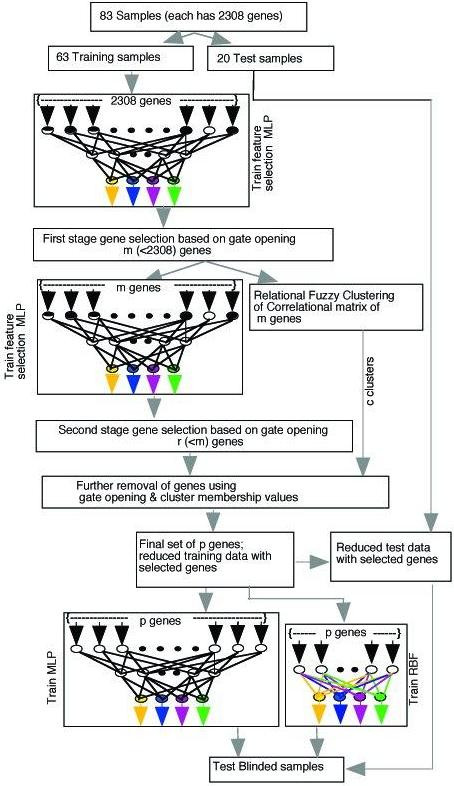
**Flow-chart of the entire process**. First the data set is divided into training and test sets. Then in the first stage, FSMLP is used to select 20 important genes. In the next stage, again FSMLP is used to select 10 useful genes from the 20 genes selected in the first stage. Now, NERFCM is applied to cluster the 20 genes selected in the first stage considering only the training data. Six clusters are found using NERFCM. The results of NERFCM and the second stage of gene selection are combined to choose just seven genes with sufficient cancer-specific signatures to distinguish between the 4 types of SRBCTs. Now different classifiers such as MLP, SVMs, RBF nets are trained using the training set with the selected 7 genes. The trained system (MLP/RBF net/SVM) is then used for classification of blind test data.

Figure 6 depicts the overall flow of the method. First we train a FSMLP with 2308 input nodes and 150 hidden nodes using the 63 training samples as mentioned above. We did not try to optimize the network size because in this stage our problem is not to find an optimal network for prediction but a set of useful genes (not necessarily the minimal set). Since the usefulness of features is not likely to depend much on the size of the network (size can, of course, influence the learning speed and the minimum where the network lands at, and hence the feature set; but we are interested in one such set), the choice of the number of hidden nodes is not a critical issue at this stage. The choice should be adequate so that the data can be learnt. So, to decide on the number of nodes we made a few trails and keeping in mind the principle of minimum description length (smaller network, less degrees of freedom, lesser chance of poor generalization) decided on 150. Based on the gate opening values in the first stage we select twenty genes as listed in Table 2. In the next stage of the procedure, as shown in Figure 6, we again use FSMLP to select ten genes from the set of 20. Now we apply the NERFCM algorithm to cluster the set of twenty genes selected in the first stage. A natural question comes : why are we not clustering the 10 genes extracted in stage two? We cluster the 20 genes for two reasons: (1) we wanted to make sure that no interesting gene in the list of twenty is excluded in the set of ten genes selected in stage two; i.e., we do not want to exclude a gene with no correlated counterpart in the list of ten genes. This check is important as our analysis is based on a limited training data. For example, if we could have found a cluster with no member from the list of 10, then we would have included one of the genes from that cluster into the final list of selected genes. (2) The other reason is that finding 5–6 clusters in a set of ten data points may not be informative. In this case the average number of points per cluster is two and hence assessing the quality of clusters would be difficult. Moreover, since the list of ten is expected to have less number of correlated genes, good clusters are not expected to be found.

The results of the relational clustering and that of the second stage of gene selection are combined to pick seven genes with sufficient cancer specific signatures to discriminate between the four types of SRBCTs. This final set of seven genes are now used to train MLPs, RBF networks and SVMs. The trained machines are then used for classification of the blind test samples. Note that, the last stage in Fig. 6 shows only MLP and RBF, but the system, as we have discussed, can be augmented with other machine learning tools like SVMs.

Since FSMLP looks at all genes together and uses the same machine learning tool that is finally used to design the classifier, our method takes into account the interaction between genes and between genes and the tool. Moreover, since we use gradient descent, genes having higher discriminating power (i.e., which can reduce error faster) would open their associated gates faster. Thus, FSMLP is likely to select a small but adequate set of genes. The relational cluster analysis further reduces the number of correlated genes and hence the selected set of genes is unlikely to have redundant ones. So, although we cannot guarantee, compared to the techniques discussed here, our method is expected to select less number of genes.

### The Online Feature Selection Net

Accurate prediction of the diagnostic category of a tumor based on gene expression data is a very important problem because this can help planning of better treatment of patients. Moreover, identification of specific gene expression patterns that are linked to metabolic characteristics which contribute to different diseases is also very important. But the very high dimensional nature of the microarray data combined with the fact that such data are usually contaminated with noise, makes such a task very difficult one. This problem involve two tasks : selection of genes and designing of the diagnostic system. Most approaches to solve these problems, view them as two separate problems ignoring the fact that a set of genes best for one type of machine learning tool may not necessarily be the best for another kind of tool. Often one gene is evaluated and removed at a time in a stepwise manner and such methods may not capture subtle non-linear interactions among a set of genes. Hence the best strategy would be to select the genes simultaneously with the main learning task, the designing of the prediction system. We call such a system, "online" system. We use an online feature selection multilayer perceptron network for gene selection and designing of the diagnostic system. This novel scheme picks up the necessary genes online when the system learns the diagnostic prediction task.

For a multilayer perceptron network, the effect of some genes can be eliminated by not allowing them into the network, i.e., by equipping each input node (hence each gene/feature) with a gate and closing the gate.

For useful genes, the associated gates should be completely opened and for a partially important genes the gates should be partially opened. Pal and Chintalapudi [9] suggested a mechanism for realizing such a gate so that *not useful *features/genes can be identified and attenuated. To model the gates we associate a gate function to each node in the input layer. Each input node computes the product of the input (expression value of a gene) and the gate function value as its output. This modulated output is passed on to the next layers of the network. The gate function should produce high values for marker genes (useful features); while for a redundant/not important gene, it should be nearly 0. The gate functions attenuate the genes before they propagate through the net, so we call these gate functions *attenuating *functions. As an attenuation function, we can use any function, *F*_*i *_:*R *→ [0,1], satisfying the following conditions : (i) It has a tunable parameter and it is differentiable with respect to the tunable parameter and (ii) it is monotonic with respect to its tunable parameter. The sigmoidal function, *F*(*m*) = 1/(1 + *e*^-*m*^), satisfies these criteria and we use it here.

Our learning philosophy is to keep all gates almost closed at the beginning of the learning (i.e., as if no gene is important) and then open the gates as required during the training. Let *F*_*i *_be the gate or attenuation function associated with the *i*^*th *^gene (input). *F*_*i *_has an argument *m*_*i *_and F′i
 MathType@MTEF@5@5@+=feaafiart1ev1aaatCvAUfKttLearuWrP9MDH5MBPbIqV92AaeXatLxBI9gBaebbnrfifHhDYfgasaacH8akY=wiFfYdH8Gipec8Eeeu0xXdbba9frFj0=OqFfea0dXdd9vqai=hGuQ8kuc9pgc9s8qqaq=dirpe0xb9q8qiLsFr0=vr0=vr0dc8meaabaqaciaacaGaaeqabaqabeGadaaakeaacuWGgbGrgaqbamaaBaaaleaacqWGPbqAaeqaaaaa@2F54@ (*m*_*i*_) is the value of derivative of the attenuation function at *m*_*i*_. Let *μ *be the learning rate of the attenuation parameter; *v *be the learning rate of the connection weights, *x*_*i *_be the *i*^*th *^input (gene); x′i
 MathType@MTEF@5@5@+=feaafiart1ev1aaatCvAUfKttLearuWrP9MDH5MBPbIqV92AaeXatLxBI9gBaebbnrfifHhDYfgasaacH8akY=wiFfYdH8Gipec8Eeeu0xXdbba9frFj0=OqFfea0dXdd9vqai=hGuQ8kuc9pgc9s8qqaq=dirpe0xb9q8qiLsFr0=vr0=vr0dc8meaabaqaciaacaGaaeqabaqabeGadaaakeaacuWG4baEgaqbamaaBaaaleaacqWGPbqAaeqaaaaa@2FB8@ be the attenuated value of *x*_*i*_, i.e., x′i
 MathType@MTEF@5@5@+=feaafiart1ev1aaatCvAUfKttLearuWrP9MDH5MBPbIqV92AaeXatLxBI9gBaebbnrfifHhDYfgasaacH8akY=wiFfYdH8Gipec8Eeeu0xXdbba9frFj0=OqFfea0dXdd9vqai=hGuQ8kuc9pgc9s8qqaq=dirpe0xb9q8qiLsFr0=vr0=vr0dc8meaabaqaciaacaGaaeqabaqabeGadaaakeaacuWG4baEgaqbamaaBaaaleaacqWGPbqAaeqaaaaa@2FB8@ = *x*_*i*_*F*(*m*_*i*_); wij0
 MathType@MTEF@5@5@+=feaafiart1ev1aaatCvAUfKttLearuWrP9MDH5MBPbIqV92AaeXatLxBI9gBaebbnrfifHhDYfgasaacH8akY=wiFfYdH8Gipec8Eeeu0xXdbba9frFj0=OqFfea0dXdd9vqai=hGuQ8kuc9pgc9s8qqaq=dirpe0xb9q8qiLsFr0=vr0=vr0dc8meaabaqaciaacaGaaeqabaqabeGadaaakeaacqWG3bWDdaqhaaWcbaGaemyAaKMaemOAaOgabaGaeGimaadaaaaa@31F6@ be the weight connecting the *j*^*th *^node of the first hidden layer to the *i*^*th *^node of the input layer; and δj1
 MathType@MTEF@5@5@+=feaafiart1ev1aaatCvAUfKttLearuWrP9MDH5MBPbIqV92AaeXatLxBI9gBaebbnrfifHhDYfgasaacH8akY=wiFfYdH8Gipec8Eeeu0xXdbba9frFj0=OqFfea0dXdd9vqai=hGuQ8kuc9pgc9s8qqaq=dirpe0xb9q8qiLsFr0=vr0=vr0dc8meaabaqaciaacaGaaeqabaqabeGadaaakeaaiiGacqWF0oazdaqhaaWcbaGaemOAaOgabaGaeGymaedaaaaa@30D2@ be the error term for the *j*^*th *^node of the first hidden layer [9].

It is straightforward to show that except for wij0
 MathType@MTEF@5@5@+=feaafiart1ev1aaatCvAUfKttLearuWrP9MDH5MBPbIqV92AaeXatLxBI9gBaebbnrfifHhDYfgasaacH8akY=wiFfYdH8Gipec8Eeeu0xXdbba9frFj0=OqFfea0dXdd9vqai=hGuQ8kuc9pgc9s8qqaq=dirpe0xb9q8qiLsFr0=vr0=vr0dc8meaabaqaciaacaGaaeqabaqabeGadaaakeaacqWG3bWDdaqhaaWcbaGaemyAaKMaemOAaOgabaGaeGimaadaaaaa@31F6@, the update rules for all weights remain the same as that for an ordinary MLP trained with backpropagation. Assuming that the first hidden layer has q nodes, the update rules for wij0
 MathType@MTEF@5@5@+=feaafiart1ev1aaatCvAUfKttLearuWrP9MDH5MBPbIqV92AaeXatLxBI9gBaebbnrfifHhDYfgasaacH8akY=wiFfYdH8Gipec8Eeeu0xXdbba9frFj0=OqFfea0dXdd9vqai=hGuQ8kuc9pgc9s8qqaq=dirpe0xb9q8qiLsFr0=vr0=vr0dc8meaabaqaciaacaGaaeqabaqabeGadaaakeaacqWG3bWDdaqhaaWcbaGaemyAaKMaemOAaOgabaGaeGimaadaaaaa@31F6@ and *m*_*i *_are:

wij,new0=wij,old0−vxiδj1F(mi)     (1)
 MathType@MTEF@5@5@+=feaafiart1ev1aaatCvAUfKttLearuWrP9MDH5MBPbIqV92AaeXatLxBI9gBaebbnrfifHhDYfgasaacH8akY=wiFfYdH8Gipec8Eeeu0xXdbba9frFj0=OqFfea0dXdd9vqai=hGuQ8kuc9pgc9s8qqaq=dirpe0xb9q8qiLsFr0=vr0=vr0dc8meaabaqaciaacaGaaeqabaqabeGadaaakeaacqWG3bWDdaqhaaWcbaGaemyAaKMaemOAaOMaeiilaWIaemOBa4MaemyzauMaem4DaChabaGaeGimaadaaOGaeyypa0Jaem4DaC3aa0baaSqaaiabdMgaPjabdQgaQjabcYcaSiabd+gaVjabdYgaSjabdsgaKbqaaiabicdaWaaakiabgkHiTiabdAha2jabdIha4naaBaaaleaacqWGPbqAaeqaaGGacOGae8hTdq2aa0baaSqaaiabdQgaQbqaaiabigdaXaaakiabdAeagjabcIcaOiabd2gaTnaaBaaaleaacqWGPbqAaeqaaOGaeiykaKIaaCzcaiaaxMaadaqadaqaaiabigdaXaGaayjkaiaawMcaaaaa@5576@

mi,new0=mi,old0−μxi(∑j=1qwij0δj1)F′(mi)     (2)
 MathType@MTEF@5@5@+=feaafiart1ev1aaatCvAUfKttLearuWrP9MDH5MBPbIqV92AaeXatLxBI9gBaebbnrfifHhDYfgasaacH8akY=wiFfYdH8Gipec8Eeeu0xXdbba9frFj0=OqFfea0dXdd9vqai=hGuQ8kuc9pgc9s8qqaq=dirpe0xb9q8qiLsFr0=vr0=vr0dc8meaabaqaciaacaGaaeqabaqabeGadaaakeaacqWGTbqBdaqhaaWcbaGaemyAaKMaeiilaWIaemOBa4MaemyzauMaem4DaChabaGaeGimaadaaOGaeyypa0JaemyBa02aa0baaSqaaiabdMgaPjabcYcaSiabd+gaVjabdYgaSjabdsgaKbqaaiabicdaWaaakiabgkHiTGGaciab=X7aTjabdIha4naaBaaaleaacqWGPbqAaeqaaOGaeiikaGYaaabmaeaacqWG3bWDdaqhaaWcbaGaemyAaKMaemOAaOgabaGaeGimaadaaOGae8hTdq2aa0baaSqaaiabdQgaQbqaaiabigdaXaaaaeaacqWGQbGAcqGH9aqpcqaIXaqmaeaacqWGXbqCa0GaeyyeIuoakiabcMcaPiqbdAeagzaafaGaeiikaGIaemyBa02aaSbaaSqaaiabdMgaPbqabaGccqGGPaqkcaWLjaGaaCzcamaabmaabaGaeGOmaidacaGLOaGaayzkaaaaaa@6099@

The gate parameters for all genes are so initialized that when the training starts *F*(*m*) is practically zero for all gates, i.e., no gene is allowed to enter the network. As the learning proceeds, gates for the genes that can reduce the error faster are opened faster. The learning of the gate function continues along with other weights of the network. At the end of the training we pick up important genes based on the values of the gate opening. The training can be stopped when the training error is reduced to zero or to an acceptable level or after a fixed number of iterations. Note that, different initializations of the network may lead to different subsets of important genes. If this happens, this indicates that there are different sets of features that can do the prediction task equally well. Since we do not use any regularization on *w*_*ij *_and *m*_*i*_, given a set of *w*_*ij *_and *m*_*i*_, there exists many sets of scaled version of connection weights and gate opening parameters with the same network behavior. However, since we use gradient descent with a low learning rate and keep all gates almost closed at the beginning of training, on termination we get a useful network without any problem. We use a small set of genes for which gates are reasonably open.

In this investigation, in the first stage we used an MLP with 2308 nodes in the input layer, 150 nodes in the hidden layer, and 4 nodes in the output layer. The network was trained till the misclassification on the training set reduced to zero or it attained a maximum number of 5000 iteration. As discussed earlier, we used the backpropagation learning algorithm for this phase. Based on the trained network, we selected twenty genes as listed in Table 2.

To decide on the number of features to be selected based on the gate opening one can proceed as follows. Let *G *be the maximum gate opening of a feature in a trial. Then select all features having gate opening more than *P*% (say *P *= 80) of *G*. Train a net with these features. If it can learn the data satisfactorily (can achieve the same or almost same level of classification accuracy as can be obtained using all features), you increase P by, say, 2%; otherwise, reduce it by 2% and repeat the process of training. This can be easily automated. If enough data are available, we can use training-validation-test partition to get better choices of such thresholds. The other alternative is to look at the gate openings. Typically useful gates are opened much more than redundant ones and based on that one can decide on a threshold. In the present case, analyzing the gate opening values we simply experimented with a few possibilities like top 18, 20 and 25 features and all sets were found to have adequate discriminative power and we decided on 20.

For a very high dimensional data set, two reasonably correlated genes x and y in conjunction with some other genes may be able to reduce the error faster, but both x and y may not be needed. Thus, this set of selected genes may contain genes which are to some extent correlated and hence redundant. If it is so, it may be possible to remove further some of them. To guard against this we do two things: (i) we use the FSMLP again on the set of twenty selected genes; and (ii) we use the non-Euclidean relational fuzzy c-means clustering algorithm (see next section) to cluster the twenty selected genes. Unlike hierarchical clustering algorithm, the NERFCM algorithm produces a set of membership values with each data point that helps us to assess how strongly a point belongs to a cluster. Moreover, different runs of NERFCM can produce different partitions which can be used to check the consistency of clusters. We want to exploit this information.

In the second phase of gene selection we apply FSMLP on the twenty genes from phase 1 and select ten genes based on the gate openings. We made many runs of MLP using the selected ten genes to ensure that this set has the required discriminating signatures. Then we use the results of NERFCM clustering to remove 3 more genes from this set of ten genes. Finally, in the third phase, an MLP is trained with seven input nodes, six nodes in the hidden layer and four output nodes. We used the Levenberg-Marquardt search method as implemented in Matlab neural network toolbox.

We have decided the desired number of clusters (c) by analyzing the consistency of partitions generated by NERFCM for different initializations and choices of c. In order to automate this process, we may need to define a cluster validity index [34,35] for relational clustering. Our broad cluster analysis guidelines are : if a cluster does not intersect the list of top ten, then select the gene with the highest gate opening value, else from the cluster members belonging to the top ten, select the one with the highest gate opening. Further, if more than one cluster member belong to the top ten, and their gate openings are very high and very close, we retain them. Although not a trivial task, such a process may be automated if we have sufficient data. In that case we can partition the data into training, validation and test sets and use the validation data to decide the thresholds on the gate opening values. The system can then be tested on the test set.

### NonEuclidean Relational Fuzzy c-means (NERFCM) Clustering of the selected genes

If *R *= [*r*_*ij*_]_*n *× *n *_is any relation satisfying the conditions :(i) *r*_*ij *_≥ 0∀_*i*,*j*_; (ii) *r*_*ii *_= 0; and (iii) *r*_*ij *_= *r*_*ji*_, we call *R *a dissimilarity relation. The relation *R *may not satisfy the triangle inequality, if it does, then it is a distance function. A dissimilarity relation *R *= [*r*_*ij*_]_*n *× *n *_is Euclidean, if there exists a set of vectors {**x**_1_, ..., **x**_*n*_} ⊂ *R*^*n*-1 ^such that *r*_*ij *_= the Euclidean distance between **x**_*i *_and **x**_*j*_; otherwise, it is non-Euclidean. In the present case, our objective is to find whether there exists some genes which are to some extent positively correlated. If there are m samples and n genes, then we have n vectors, each in *R*^*m*^, {**x**_1_, ..., **x**_*n*_} ⊂ *R*^*m*^. Note that, we are not talking about distance between such vectors because two highly correlated vectors may have a very high distance. Even two positively correlated genes and two negatively correlated genes may result in similar distances. Hence, we compute the Pearson's correlation coefficient between pairs of vectors : *R *= [*r*_*ij*_], *r*_*ij *_= correlation coefficient between gene *i *and gene *j*, i.e., between **x**_*i *_and **x**_*j*_. This R can have both positive and negative values and hence is not a dissimilarity relation. To convert it to a dissimilarity relation we use the following transformation : *R *= **1 **- *R*, i.e., *r*_*ij *_= 1 - *r*_*ij*_. This transformation will make *r*_*ii *_= 0, *r*_*ij *_≥ 0. The symmetry property of R will be maintained and the negatively correlated vectors will show higher dissimilarity while the positively correlated vectors will show lower dissimilarity. This is a valid dissimilarity relation, not necessarily Euclidean. We want to cluster this relation into c clusters. The NERFCM algorithm finds a fuzzy partition matrix *U *= [*u*_*i,k*_]_*c*,*n*_. Here *u*_*i,k *_gives the membership value (the degree) with which **x**_*k *_belongs to the *i*^*th *^cluster, *u*_*i*,*k *_≥ 0; ∑i=1i=cui,k=1
 MathType@MTEF@5@5@+=feaafiart1ev1aaatCvAUfKttLearuWrP9MDH5MBPbIqV92AaeXatLxBI9gBaebbnrfifHhDYfgasaacH8akY=wiFfYdH8Gipec8Eeeu0xXdbba9frFj0=OqFfea0dXdd9vqai=hGuQ8kuc9pgc9s8qqaq=dirpe0xb9q8qiLsFr0=vr0=vr0dc8meaabaqaciaacaGaaeqabaqabeGadaaakeaadaaeWaqaaiabdwha1naaBaaaleaacqWGPbqAcqGGSaalcqWGRbWAaeqaaaqaaiabdMgaPjabg2da9iabigdaXaqaaiabdMgaPjabg2da9iabdogaJbqdcqGHris5aOGaeyypa0JaeGymaedaaa@3CDD@. This membership values help us to assess how compact a cluster is. The NERFCM algorithm [10] converts a non-Euclidean relation to an Euclidean one using a transformation, known as *β*-transformation and then uses an iterative algorithm to estimate the membership matrix *U *[36]. The beta transformation is *R *⇒ *R*_*β *_= *R *+ *β *(*M *- *I*_*n*_), where *M*_*i,j *_= 1∀*i*,*j*, *I*_*n *_is the *n *× *n *identity matrix and *β *is suitably chosen scaler.

### The RBF network and Support Vector Machines

For the Radial basis function network, we have used the Matlab neural network toolbox and used the function *newrb*. We did not use a fully optimized network because we have used the same spread for all Gaussian nodes.

The SVM [29] maximizes the margin of separation between two classes to find a separating hyperplane *f*(**x**) = ∑i=1i=pwixi+b
 MathType@MTEF@5@5@+=feaafiart1ev1aaatCvAUfKttLearuWrP9MDH5MBPbIqV92AaeXatLxBI9gBaebbnrfifHhDYfgasaacH8akY=wiFfYdH8Gipec8Eeeu0xXdbba9frFj0=OqFfea0dXdd9vqai=hGuQ8kuc9pgc9s8qqaq=dirpe0xb9q8qiLsFr0=vr0=vr0dc8meaabaqaciaacaGaaeqabaqabeGadaaakeaadaaeWaqaaiabdEha3naaBaaaleaacqWGPbqAaeqaaOGaemiEaG3aaSbaaSqaaiabdMgaPbqabaaabaGaemyAaKMaeyypa0JaeGymaedabaGaemyAaKMaeyypa0JaemiCaahaniabggHiLdGccqGHRaWkcqWGIbGyaaa@3DFF@ = **w'x **+ *b*, where *p *is the number of genes. The SVM finds **w **= (*w*_1_, *w*_2_, ..., *w*_*p*_)*' *minimizing 12
 MathType@MTEF@5@5@+=feaafiart1ev1aaatCvAUfKttLearuWrP9MDH5MBPbIqV92AaeXatLxBI9gBaebbnrfifHhDYfgasaacH8akY=wiFfYdH8Gipec8Eeeu0xXdbba9frFj0=OqFfea0dXdd9vqai=hGuQ8kuc9pgc9s8qqaq=dirpe0xb9q8qiLsFr0=vr0=vr0dc8meaabaqaciaacaGaaeqabaqabeGadaaakeaadaWcaaqaaiabigdaXaqaaiabikdaYaaaaaa@2E9E@||**w**||^2 ^subject to the constraints *y*_*i*_(**w'x **+ *b*) ≥ 1 ∀ *i *= 1, ..., *n*, where *y*_*i *_= +1 *or *-1 depending on the class. When the training data are not linearly separable, SVM minimizes 12
 MathType@MTEF@5@5@+=feaafiart1ev1aaatCvAUfKttLearuWrP9MDH5MBPbIqV92AaeXatLxBI9gBaebbnrfifHhDYfgasaacH8akY=wiFfYdH8Gipec8Eeeu0xXdbba9frFj0=OqFfea0dXdd9vqai=hGuQ8kuc9pgc9s8qqaq=dirpe0xb9q8qiLsFr0=vr0=vr0dc8meaabaqaciaacaGaaeqabaqabeGadaaakeaadaWcaaqaaiabigdaXaqaaiabikdaYaaaaaa@2E9E@||**w**||^2 ^+ *C *∑i=1nξi
 MathType@MTEF@5@5@+=feaafiart1ev1aaatCvAUfKttLearuWrP9MDH5MBPbIqV92AaeXatLxBI9gBaebbnrfifHhDYfgasaacH8akY=wiFfYdH8Gipec8Eeeu0xXdbba9frFj0=OqFfea0dXdd9vqai=hGuQ8kuc9pgc9s8qqaq=dirpe0xb9q8qiLsFr0=vr0=vr0dc8meaabaqaciaacaGaaeqabaqabeGadaaakeaadaaeWaqaaGGaciab=57a4naaBaaaleaacqWGPbqAaeqaaaqaaiabdMgaPjabg2da9iabigdaXaqaaiabd6gaUbqdcqGHris5aaaa@36AA@ subject to the constraints *y*_*i*_(**w'x **+ *b *+ *ξ*_*i*_) ≥ 1 ∀ *y*_*i *_= +1; *y*_*i*_(**w'x **+ *b *- *ξ*_*i*_) ≥ 1 ∀ *y*_*i *_= -1; *ξ*_*i *_≥ 0, ∀ *i*. The *ξ*_*i*_'s are the slack variables. More often than not, the inputs are implicitly projected into a high dimensional space to make them more separable and the separating hyperplane is then found in the projected space. To realize this projection, different kernels can be used. In this investigation, we have used the Gaussian (also known as RBF) kernels with same spread for all cases. All experiments are done using the SVM tool available at [37].

## Authors' contributions

All authors contributed significantly to the investigation. NRP and SIA coordinated the study and involved in the formulation of methodology and the analysis scheme adopted here. AS and NRP made the software implementation and did the computations. KA and AS involved in the interpretation of the results and provided biological inputs and insights. KA and NRP wrote the first draft. All authors have read and approved the final manuscript.
